# Effects of the Heterodimeric Neurotoxic Phospholipase A_2_ from the Venom of *Vipera nikolskii* on the Contractility of Rat Papillary Muscles and Thoracic Aortas

**DOI:** 10.3390/toxins16020100

**Published:** 2024-02-10

**Authors:** Alexey Averin, Vladislav Starkov, Victor Tsetlin, Yuri Utkin

**Affiliations:** 1 Institute of Cell Biophysics, Federal Research Center “Pushchino Scientific Center of Biological Research”, Pushchino Branch, Russian Academy of Sciences, Pushchino 142290, Russia; averinas82@gmail.com; 2 Shemyakin–Ovchinnikov Institute of Bioorganic Chemistry, Russian Academy of Sciences, Moscow 117997, Russia; vladislavstarkov@mail.ru (V.S.); victortsetlin3f@gmail.com (V.T.)

**Keywords:** aorta, contraction, heart, heterodimeric phospholipase A_2_, Nikolsky’s viper, papillary muscle, venom

## Abstract

Phospholipases A_2_ (PLA_2_s) are a large family of snake toxins manifesting diverse biological effects, which are not always related to phospholipolytic activity. Snake venom PLA_2_s (svPLA_2_s) are extracellular proteins with a molecular mass of 13–14 kDa. They are present in venoms in the form of monomers, dimers, and larger oligomers. The cardiovascular system is one of the multiple svPLA_2_ targets in prey organisms. The results obtained previously on the cardiovascular effects of monomeric svPLA_2_s were inconsistent, while the data on the dimeric svPLA_2_ crotoxin from the rattlesnake *Crotalus durissus terrificus* showed that it significantly reduced the contractile force of guinea pig hearts. Here, we studied the effects of the heterodimeric svPLA_2_ HDP-1 from the viper *Vipera nikolskii* on papillary muscle (PM) contractility and the tension of the aortic rings (ARs). HDP-1 is structurally different from crotoxin, and over a wide range of concentrations, it produced a long-term, stable, positive inotropic effect in PMs, which did not turn into contractures at the concentrations studied. This also distinguishes HDP-1 from the monomeric svPLA_2_s, which at high concentrations inhibited cardiac function. HDP-1, when acting on ARs preconstricted with 10 μM phenylephrine, induced a vasorelaxant effect, similar to some other svPLA_2_s. These are the first indications of the cardiac and vascular effects of true vipers’ heterodimeric svPLA_2_s.

## 1. Introduction

Snake venoms are cocktails of proteins, peptides, low-molecular-weight organics, and salts [1]. To be effective, they target the most vitally important systems of prey organisms, with the cardiovascular system being one of them [2,3].

Among snake venoms, the most famous for their cardiotoxicity are cobra venoms, which contain cardiotoxins that produce concentration-dependent effects resulting in papillary muscle (PM) contractures and aortic ring (AR) contractions [4]. Although the venoms of snakes from other genera contain no cardiotoxins [5,6,7], they disturb the function of the heart and blood vessels. Thus, the venom of the coral snake *Micrurus lemniscatus lemniscatus* caused the relaxation of endothelium-intact aortic strips preconstricted with phenylephrine and a transient increase in the contractile force of the atria without affecting the frequency of their contractions [8]. The relaxation of preconstricted aortas was observed after the application of the venoms from *Montivipera bornmuelleri* [9] and *Lachesis acrochorda* [10], with the effect being concentration-dependent. In isolated rat right atrium preparations, the venom of *L. acrochorda* concentration-dependently (1–1000 μg/mL) increased the spontaneous contraction frequency [10]. The venom of *Bothrops jararacussu* produced concentration-dependent contracture in isolated rat right atria that was not reversed by washing. The treatment of the venom with the phospholipase A_2_ (PLA_2_) inhibitor p-bromophenacyl bromide abolished the venom-induced contracture. These results indicate that PLA_2_ is involved in the adverse effects of the venom on rat atria [11]. In isolated rat hearts, the venom of the nose-horned viper, *Vipera ammodytes ammodytes*, produced a concentration-dependent decrease in contractility and coronary flow. The transient increase in heart rate was followed by a significant decrease, and at a venom concentration of 150 μg/mL, irreversible asystolic cardiac arrest was observed [12]. In isolated rat hearts, a significant disturbance of myocardial functions was found after envenomation by an intramuscular injection of *V. aspis* venom [13]. The venom of Nikolsky’s viper, *V. nikolskii*, studied in the present work also affects the cardiovascular system. For example, signs of heart failure were observed in mice after the subcutaneous injection of *V. nikolskii* venom [14], and a non-lethal bite of a human by *V. nikolskii* resulted in peripheral vasospastic disorder [15]. Interestingly, the venoms of the three vipers mentioned above contain so-called heterodimeric PLA_2_s, which are discussed below.

Thus, snake venoms produce cardiovascular effects [8,9,10], and PLA_2_s are among the toxins responsible for this activity. In the venoms of snakes, PLA_2_s are some of the main components and manifest various pharmacological effects [16], including, but not limited to, neurotoxic, myotoxic, hemolytic, anticoagulant, and cytotoxic activities. Cardiac and vascular effects have been described for some PLA_2_s, but the data are not as extensive for other activities [17,18,19,20]. PLA_2_s from snake venoms belong to the so-called secreted PLA_2_s, and typically, these PLA_2_s are small proteins with a molecular weight in the range of 13–14 kDa [16,21]. Secreted PLA_2_s are classified into 10 groups and 18 subgroups [22]. Snake venom PLA_2_s are included in groups I and II [21].

In addition to this classification, based mainly on the differences in amino acid sequences, PLA_2_s may possess different quaternary structures. Thus, most of these enzymes are single-chain toxins with six or seven disulfide bridges. The PLA_2_s manifesting cardiovascular effects [17,18,19,20] belong to this class. Another class includes PLA_2_s that consist of two homologous noncovalently bound subunits, at least one of which possesses phospholipolytic activity. For example, these are crotoxin and related toxins from pit vipers of the genus *Crotalus*, which are heterodimers of a basic enzymatically active subunit and an inactive acidic subunit [23]. Similar heterodimers are also present in the venoms of several species of snakes from the genus *Vipera*. Examples include vipoxin from *Vipera ammodytes ammodytes* [24] and HDP-1 from *V. nikolskii* [25].

In the venom of Nikolsky’s viper (*V. nikolskii*) studied in the present work, PLA_2_s dominate, comprising about 65% of the venom [26]. PLA_2_s are represented by two heterodimeric toxins, HDP-1 and HDP-2 [25], which consist of two homologous subunits bound noncovalently. One subunit is an enzymatically active basic protein with a molecular mass of about 13.8 kDa, and the other one is an inactive acidic protein with a molecular mass of about 13.6 kDa. In the acidic subunit, the histidine residue at position 48 (amino acid numbering according to Renetseder et al. [27]) in the active center is replaced by glutamine. HDP-1 and HDP-2 manifest anticoagulant and anti-platelet activity. At the frog neuromuscular junction, they act presynaptically, affecting neuromuscular transmission [25]. The amino acid sequences of HDP-1 and HDP-2 are homologous to those of the heterodimeric PLA_2_s vaspin from *V. aspis* and vipoxin from *V. ammodytes*. Thus, HDP-1 and HDP-2 belong to the PLA_2_ class, which includes enzymes consisting of two homologous subunits linked noncovalently, with at least one of the subunits possessing phospholipolytic activity. Their subunits are of group IIA, containing seven disulfide bridges and C-terminal extensions of seven amino acid residues [22].

The structural diversity of snake venom PLA_2_s (svPLA_2_s) suggests a vast variety of physiological effects; however, among the heteromeric PLA_2_s, only controversial data on the cardiovascular effects of crotoxin are available. Thus, in isolated guinea pig hearts perfused using the Langendorff method, crotoxin caused a marked decrease in contractile force without significantly reducing the heart rate [28]. High concentrations of crotoxin (more than 5 μM) completely blocked the cardiac action potential as well as the beating of neonatal rat cardiomyocytes [29]. Surprisingly, lower concentrations (2–4 μM) of crotoxin led to the strong potentiation of L-type Ca^2+^ currents [29], but the reasons for this discrepancy still need to be studied. On the other hand, in a more recent study, no effects were observed 12 h after treating rat hearts with crotoxin [30]. The data on crotoxin’s vascular effects are also inconsistent; while in one work, it was shown that crotoxin induced contractions in Sprague Dawley rat ARs both with and without an endothelium [31], no effect of this toxin in AR assays was observed in another study [32]. To clarify this issue, we decided to study the effects of the heterodimeric PLA_2_ HDP-1, which is homologous to crotoxin.

The aim of this work was to determine what effects the heterodimeric neurotoxic PLA_2_ from the venom of *V. nikolskii* can have on the contractility of rat PMs and thoracic aortas. Both heterodimeric PLA_2_s present in *V. nikolskii* venom exhibited very similar enzymatic, coagulant, and neurotoxic properties, and their amino acid sequences differed by only two positions [25]. The glutamine residue at position 110 and lysine at position 118 in the basic subunit of HDP-1 were replaced by lysine and arginine, respectively, in HDP-2, while there was no difference in the acidic subunit. We chose HDP-1 for our research as it showed slightly higher biological activities [25]. We decided to study its effect on PM contractility because this allows for the assessment of many aspects of myocardial tissue physiology [33]. The PMs are an experimental model with completely preserved intercellular and intracellular structures, making them particularly useful for testing the functional effects of various bioactive compounds. Thoracic ARs were chosen as the object of study for the selected toxin’s vascular effects, as they are one of the generally accepted models for assessing both vasoconstrictive and vasorelaxant effects [34]. It was found that in the PMs, HDP-1 produced a long-term, stable, positive inotropic effect over a wide range of concentrations, which did not turn into contractures. This distinguishes it from the monomeric svPLA_2_s, which at high concentrations inhibited cardiac function. In ARs preconstricted with 10 μM phenylephrine, HDP-1 induced a vasorelaxant effect, similar to other venom PLA_2_s.

## 2. Results

### 2.1. Effects of HDP-1 on Rat PM Contractility

**Figure 1:**
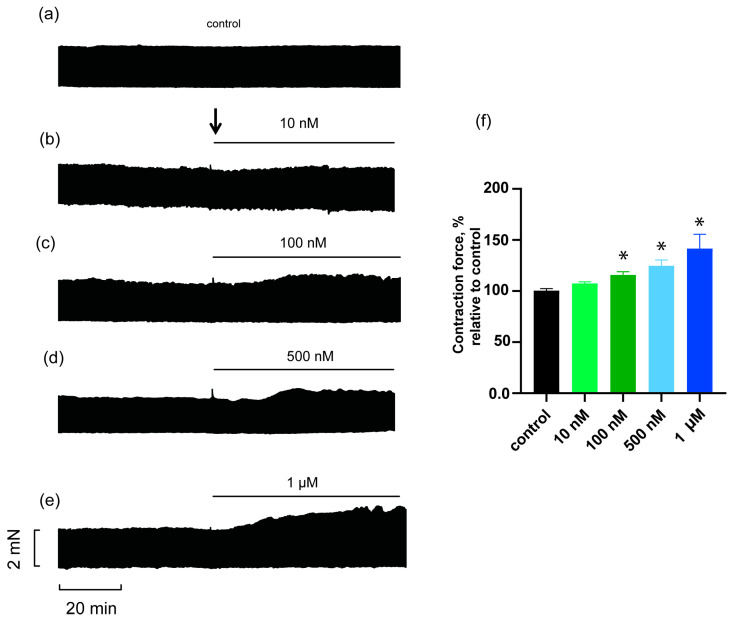
Effect of different concentrations of HDP-1 on the contraction force of the papillary muscles (PMs). Representative traces show the control ((**a**) *n* = 5; here and in the next figures, this is the number of PMs) and the effects of HDP-1 at concentrations of 10 nM ((**b**) *n* = 5), 100 nM ((**c**) *n* = 5), 500 nM ((**d**) *n* = 6), and 1 μM ((**e**) *n* = 6). The arrow indicates the time point at which HDP-1 was added. (**f**) Quantitative data, where the ordinate shows the force of contraction at 0.3 Hz normalized to that obtained before the addition of HDP-1. Similar to the experimental groups, the contraction force in a separate control group (*n* = 5) was recorded 30 min after the start of the measurement. Data are presented as the mean ± SEM. * *p* < 0.05.

HDP-1 was prepared exactly as described [25] (Figure S1). The structure of the isolated protein was confirmed with high-resolution mass spectrometry (Figure S2). To study the effect of HDP-1 on the heart, rat PMs were used. The forces of the stimulated muscle contractions were recorded at different concentrations of HDP-1 added to the organ bath solution. It was found that HDP-1 at a concentration of 10 nM induced a slight increase in contractile force by 7 ± 2% (Figure 1b,f). As the concentration of HDP-1 increased, the force of the contractions grew. An HDP-1 concentration of 100 nM led to a considerable increase in contractile force by 16 ± 3% (Figure 1c,f), and at 500 nM and 1 μM, the increase was equal to 28 ± 6% (Figure 1d,f) and 42 ± 14 (Figure 1e,f), respectively. No contracture was observed in any of the experiments. It should also be noted that none of the studied concentrations had an effect on the time parameters of the contractions, such as the time to peak tension (Table S1, Figure S3) and the time to 50 and 95% relaxation (Table S1).

### 2.2. Frequency Dependence of HDP-1′s Effect on Force of PM Contraction

**Figure 2:**
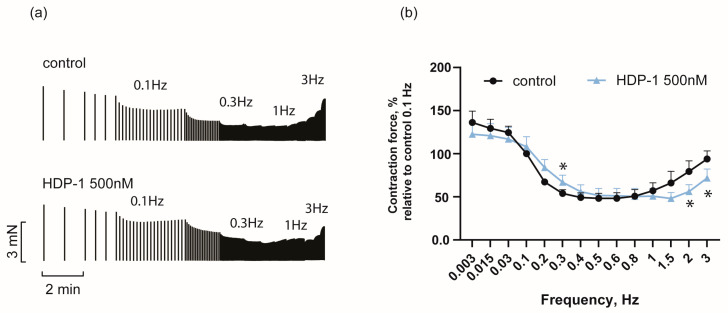
Effect of HDP-1 (500 nM) on the force–frequency relation of the PMs. (**a**) Representative traces show the dependence of the contraction force on the stimulation frequency. The stimulation rate was increased stepwise to 0.1, 0.2, 0.3, 0.4, 0.5, 0.6, 0.8, 1.0, 1.5, 2.0, and 3.0 Hz. (**b**) Statistical data, where the ordinate shows the force of contraction normalized to the force of contraction obtained at 0.1 Hz in the control. Data are presented as the mean ± SEM. *n* = 6. * *p* < 0.05.

Because the pacing frequency itself is a modulator of cardiac contraction force [35], and given the fact that some PLA_2_ effects may be frequency-dependent [17], we carried out the study using a wide frequency range (from 0.003 to 3 Hz). For this experiment, an HDP-1 concentration of 500 nM was chosen since, at this concentration, an increase in the force of contractions is clearly visible, and the amount of protein required to perform the experiment is relatively small. From the data presented in Figure 2, it can be seen that in the frequency range from 0.003 to 0.1 Hz, the PLA_2_ effect is practically absent. At 0.2–0.3 Hz, a positive inotropic effect of 24 ± 15% is observed (Figure 2); then, with increasing stimulation frequency, the effect weakens and becomes negative after 1 Hz, decreasing by 29 ± 11% (Figure 2) at a stimulation frequency of 2 Hz.

### 2.3. Effect of HDP-1 on Post-Rest Potentiation of PMs

**Figure 3:**
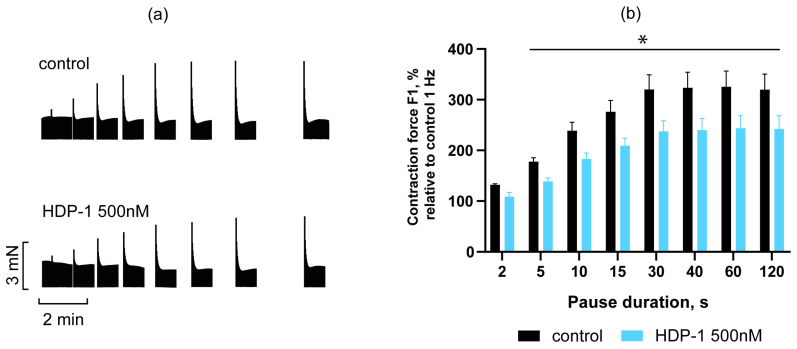
Effect of HDP-1 (500 nM) on post-rest potentiation of PMs. (**a**) Representative traces show the dependence of the contraction force on the duration of the pause. (**b**) Statistical data, where the ordinate shows the force of contraction normalized to the force of contraction obtained at 0.1 Hz in the control. Data are presented as the mean ± SEM, *n* = 5. * *p* < 0.05.

The effect of HDP-1 on the post-rest potentiation of the PMs was studied at a concentration of 500 nM, and the data obtained are shown in Figure 3. As can be seen, for all pause durations studied, with the exception of 2 s, the potentiation effect was significantly reduced by exposure to 500 nM HDP-1 (Figure 3). Moreover, already with a pause duration of 10 s, it decreased by 23 ± 5%, reaching a maximum decrease of 26 ± 7% with a 30 s pause.

### 2.4. Vasorelaxant Effects of HDP-1 on Rat ARs

**Figure 4:**
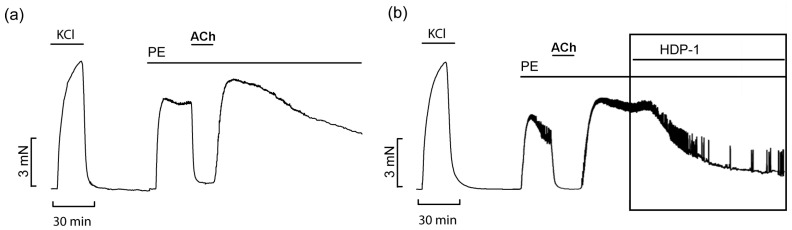
Schematic illustration of the experiments on the contraction of the ARs. KCl, isotonic solution with 80 mM KCl; ACh, 10 µM acetylcholine; PE, 10 μM phenylephrine. (**a**) Control. In (**b**), HDP-1 was added at a concentration of 100 nM. Horizontal lines indicate the presence of the corresponding reagent in the washing solution. The rectangle shows the registered relaxant effect of HDP-1 at the concentration applied (10 nM to 1 μM).

To study the possible vasorelaxant effects of HDP-1, experiments on rat ARs preconstricted with 10 μM phenylephrine (PE) were carried out. ARs with an intact endothelium were used. A schematic illustration explaining the experimental details is shown in Figure 4. The maximal relaxation induced by acetylcholine (ACh) indicates the integrity of the endothelium. Thirty minutes after the administration of HDP-1, the relaxation level was calculated relative to the tension level registered after ACh washout with a PE (10 μM)-containing solution for thirty minutes; this value was taken as 0% relaxation.

**Figure 5:**
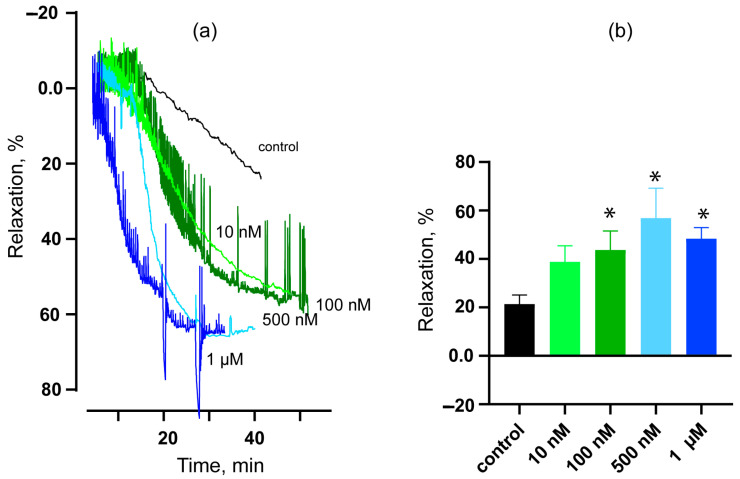
Influence of HDP-1 on tension of ARs preconstricted with 10 μM PE. (**a**) Representative traces showing the control (*n* = 9) and the effects of HDP-1 at concentrations of 10 nM (*n* = 6), 100 nM (*n* = 7), 500 nM (*n* = 4), and 1 μM (*n* = 5), where n is the number of ARs studied. (**b**) Quantitative data, where the ordinate shows the level of relaxation. Data are presented as the mean ± SEM. * *p* < 0.05 compared to the control. In the control experiment, no HDP-1 was added.

Vasorelaxant effects were observed at different concentrations of HDP-1. Thus, at an HDP-1 concentration of 10 nM, the vasorelaxant effect was equal to 38 ± 7%, although it was not significantly different from the control value of 21 ± 4% (Figure 5). As the concentration increased, the effect showed a tendency to intensify, and it reached 44 ± 8% at 100 nM, 57 ± 12% at 500 nM, and 48 ± 5% at 1 μM (Figure 5). Although the difference with the control for these concentrations was statistically significant, no significant differences were detected between the effects of different concentrations of HDP-1.

## 3. Discussion

HDP-1 is a heterodimeric PLA_2_ exhibiting biological properties shared by many svPLA_2_s [19]. In the present work, we investigated the influence of HDP-1 on the contractility of rat PMs and thoracic aortas. Experiments were carried out at 30 °C. The main reason for choosing this temperature was to avoid possible ischemic tissue damage due to the thickness of the PM preparations [36] since saline cannot completely replace blood in ex vivo experimental conditions. It has also been shown [37] that at temperatures close to 30 °C in rat trabeculae, the basic physiological reactions, such as the force–frequency relationship and the frequency-dependent acceleration of relaxation, are very similar to those recorded at physiological temperatures. It was found that the application of HDP-1 to PMs produced an inotropic effect. At a stimulation frequency of 0.3 Hz, this effect was positive and concentration-dependent. At an HDP-1 concentration of 1 μM, it reached about 40% of the control. It was previously shown that an acidic PLA_2_ (OHV A-PLA_2_) from the venom of *Ophiophagus hannah* caused a positive chronotropic effect on isolated rat right atria, and on isolated rat left atria and PMs at concentrations of 0.18–1.45 μM, it produced a positive inotropic effect, followed by contracture [18]. No contracture was observed at any of the HDP-1 concentrations up to 1 μM tested in our work; however, we cannot exclude this effect at higher HDP-1 concentrations. It should be noted that concentration-dependent muscle contracture was produced in isolated rat right atria by the venom of *B. jararacussu* [11]. As this effect was abolished by the specific PLA_2_ inhibitor p-bromophenacyl bromide, the authors ascribed the observed contracture to the action of PLA_2_.

On the other hand, studies on PLA_2_ from the venom of *N. nigricollis* showed that it produced cardiovascular effects through a mechanism that did not depend on phospholipid hydrolysis [38,39]. The conclusion that phospholipolytic activity is not necessary for cardiovascular effects is supported by studies of phospholipases from the venom of *Agkistrodon piscivorus piscivorus*, basic Asp-49 PLA_2_ and its homolog basic Lys-49 PLA_2_, which is enzymatically inactive [19]. In an isolated ventricle strip of the heart, both enzymes were practically equipotent [19]. Further, the cardiac effects of ammodytin L, a basic PLA_2_ homolog, in which Asp-49 in the active center is changed to Ser, were studied using a Langendorff model of an isolated perfused rat heart [20]. The application of ammodytin L significantly caused an increase in diastolic pressure and a reduction in developed left ventricular pressure, systolic left ventricular pressure, coronary flow, and heart rate [20]. These data confirm the hypothesis that enzymatic activity is not necessary for cardiovascular effects.

Among the cardiotoxic effects that were detected in preparations of isolated atria, the basic PLA_2_ from *N. nigricollis* decreased the amplitude of contraction, increased the time to the peak force of contraction, and prolonged the latency to the initiation of contraction [17]. In our experiments, such effects were absent, and only at an HDP-1 concentration of 500 nM we did observe a tendency towards growth in all the studied time parameters of contraction, but this tendency was not statistically significant.

Over a wide range of concentrations, HDP-1, unlike other svPLA_2_s, including crotoxin, caused a long-lasting, time-sustained, inotropic effect in PMs that did not turn into contractures. As can be seen in Figure 2, the inotropic effect of HDP-1 is frequency-dependent. Differences in the mean values are seen at almost all frequencies including 0.2 and 0.4 Hz. Indeed, they are statistically significant only at three points, and the significance is achieved at the points of the greatest difference in contraction forces. The most pronounced increase is observed at a stimulation frequency of 0.3 Hz. It is known that the myocardium of mice and rats is characterized by a specific biphasic-type force–frequency relationship [40]. In this case, the force of contraction on the descending branch mainly depends on the release of calcium ions from the sarcoplasmic reticulum, while at the same time, the ascending branch is strongly dependent on L-type Ca^2+^ channels [41]. The frequency of 0.3 Hz is in the center of the frequency range, and the increase in this region is likely due to a combination of several mechanisms. The sustained positive inotropic effect may have several explanations. Thus, PLA_2_ has been shown to inhibit Na-K ATPase [42], which, by analogy with ouabain, may have a positive inotropic effect. Additional sources of increased contraction force could be an increase in SERCA2a activity; however, such a change would lead to an acceleration of the contraction kinetics, as shown in experiments on transgenic mice with enhanced SERCA function [43,44], and an increase in the absolute values of the rest effect [43], which was not observed in our experiments. Another potential source of increased contractility may be an increase in L-type Ca^2+^ currents; however, in our experiments, for stimulation frequencies above 1 Hz, at which the contribution of L-type Ca^2+^ currents to the activation of contraction is highest [45], a slight decrease in contraction force was seen. The reason for the negative inotropic response may be either a decrease in the Ca^2+^ currents or their frequency-dependent activation, as well as a decrease in the activity of SERCA2a, which also contributes to contractility at frequencies close to physiological ones [46]; however, an increase in the force of contractions can be caused not only by the direct activation of L-type Ca^2+^ channels but also by changes in the activity of various types of potassium channels, which can indirectly change the increase in the entry of extracellular Ca^2+^ through both L-type Ca^2+^ channels and NCX [47]. Further investigations are needed to accurately establish the mechanisms underlying the effects we found.

Many toxins are known to affect the activities of the sarcoplasmic reticulum (SR) of myocardial cells [2]. In this regard, we investigated this aspect of HDP-1’s action by analyzing the post-rest effect, which is known to be a qualitative indicator of the Ca^2+^ content in the SR. This effect arises because introducing a pause in rhythmic stimulation causes potentiation of the first contraction after the pause. Typically, it is the ratio of the strength of the first contraction after a pause to the strength of the rhythmic contractions that is used as an indicator of the post-rest effect [48]. Therefore, the observed decrease in the rest effect of PMs by HDP-1 indicates a decrease in the Ca^2+^ content in the SR. There may be several reasons behind this, e.g., increased leakage from the SR through ryanodine receptors. This may also be caused by a decrease in SERCA2a activity, as has been shown for PLA_2_ from *B. jararcussu* venom [49], although this is likely not possible due to the absence of changes in the contraction kinetics mentioned above. Other mechanisms may also be involved, but further studies are needed to identify them.

Considering the action of snake venom PLA_2_s on blood vessels and on aortas in particular, it should be noted that both vasorelaxant and vasoconstrictive effects have been described in the literature. For instance, in rat ARs preconstricted with noradrenaline, acidic PLA_2_ from *V. russelli* venom induced relaxation in a concentration-dependent manner [41]. The application of the effectors of the arachidonic acid cascade and the guanylate cyclase inhibitor showed that in the rat aorta, the relaxation induced by PLA_2_ is partially mediated by lipoxygenase products and cyclic GMP [41]. Similarly, two PLA_2_s from *O. scutellatus* venom induced a relaxant effect in mesenteric arteries with an intact endothelium [50]. Interestingly, in arteries without an endothelium, the effect of one PLA_2_ was significantly reduced, while the relaxation evoked by another was not significantly affected [50]. The authors suggested that these PLA_2_s induced vascular relaxation through the release of dilator autacoids, which could be nitric oxide, prostaglandins, and some others [51]. In our work, the effect of HDP-1 on endothelium-intact aortas was studied. The observed relaxant effect of HDP-1 is in good agreement with the literature data, which means that the molecular mechanisms discussed above for other PLA_2_s may be involved in HDP-1’s action.

However, it should be noted that some snake venom PLA_2_s could exert vasoconstrictive effects. For instance, AhV_aPA, the PLA_2_ from the venom of *A. halys pallas*, induced a further contractile response of about 20% in mouse thoracic ARs preconstricted with 60 mM K^+^ [52]. Later, it was shown that AhV_aPA treatment with *p*-bromophenacyl bromide, a specific PLA_2_ inhibitor, did not significantly reduce the vasoconstrictive effect. This result indicates that the contractile response is independent of the phospholipolytic activity [53]. The authors suggested that this effect could be produced by Ca^2+^ released from the SR. Crotoxin was also shown to induce contractions in rat ARs both with and without an endothelium [31]. It was shown that indomethacin blocked crotoxin’s effect, while *N*^ω^-nitro-L-arginine was ineffective. The authors hypothesized that the activation of cyclooxygenase by crotoxin underlies the observed contractile response. This response is different from the relaxant effect of HDP-1 found in our work. The rationale for this difference is unclear. It might be explained by the nature of the PLA_2_ or the experimental conditions. This is a limitation of our work and of all other works where a single PLA_2_ is investigated. To address this uncertainty, we plan to study one more heterodimeric PLA_2_ along with a monomeric homolog.

The systemic effects of snake PLA_2_s usually include bradycardia and hypotension, which may be a consequence of their direct action on the heart and blood vessels [46,54]. As discussed above, the cardiac and vascular effects of some PLA_2_s are not related to phospholipolytic activity [20,38,39]. In HDP-1, one subunit exhibits enzymatic activity, while the other one is inactive. It would be interesting to know which subunit is responsible for the cardiovascular effects discovered in this work. Our future research goal is to isolate individual subunits and study their cardiac and vascular effects. Interestingly, in vipoxin, a very close homolog of HDP-1, the enzymatically inactive subunit is practically non-toxic to mice [55]. If this HDP-1 subunit manifests effects similar to those of heterodimeric HDP-1, it may be considered as a basis for the design of cardiostimulating and/or vasorelaxant drugs. Moreover, it is possible that some short fragment of the amino acid sequence may reproduce the observed effects of HDP-1, and the discovery of such a fragment would provide a direction for further research.

## 4. Conclusions

The effects of the heterodimeric PLA_2_ HDP-1 from the viper *V. nikolskii* on PM contractility and the relaxation of ARs were studied. Over a wide range of concentrations, HDP-1 produced in PMs a long-term, stable, positive inotropic effect, which did not turn into contractures. This distinguishes it from the monomeric svPLA_2_s, which, at high concentrations, inhibited cardiac function. HDP-1, when acting on ARs preconstricted with 10 μM phenylephrine, induced a vasorelaxant effect, similar to some other svPLA_2_s. These are the first indications of the cardiac and vascular effects of true vipers’ heterodimeric svPLA_2_s. Although we were unable to find evidence of damage to the cardiovascular system as a result of Nikolsky’s viper bites, it should be kept in mind that such complications are quite possible. On the other hand, the positive inotropic and vasorelaxant effects of HDP-1 reported here can serve as a basis for the design of new medications.

## 5. Materials and Methods

### 5.1. Materials

HDP-1 was isolated from *V nikolskii* viper venom according to a previously described procedure [25]. The venom was obtained from vipers kept in captivity, as in [56]. Glucose and inorganic salts were acquired from Merck KgaA (Darmstadt, Germany). All other reagents acquired from a local supplier were of analytical grade or higher purity.

### 5.2. Animal Handling

Adult male Wistar rats (3–4 months old, 300–350 g body weight) were used in the present work. Every effort was made to minimize animal suffering, and all operations were performed under sodium pentobarbital anesthesia (50 mg/kg i.p.). From each animal, 1 to 3 PMs and 2 to 4 ARs were prepared. The total number of used animals was 16. This study did not involve endangered or protected species and was performed in accordance with Directive 2010/63/EU [57] of the European Parliament. All experimental procedures were approved by the Biological Safety and Ethics Committee of the Institute of Cell Biophysics of the Federal Research Center “Pushchino Scientific Center of Biological Research,” Pushchino Branch, Russian Academy of Sciences. Instructions for the use of laboratory animals at the Institute of Cell Biophysics № 57 of 30 December 2011.

### 5.3. Contractility of PMs

PMs were cut from the right ventricle of anesthetized rat hearts. To study their mechanical activity, an automated instrument consisting of a personal computer and an L-154 ADC/DAC board (L-Card, Moscow, Russia) were used. The mechanical activity of the PMs was recorded using a 6 × 2M mechanotron. To calibrate the system, a load of 1 g was applied. The consistency of the recordings was checked before each experiment. Measurements of the isometric force of PM contractions were performed in oxygenated (95% O_2_/5% CO_2_) Tyrode solution containing the following (in mM): NaCl, 135; KCl, 4; MgCl_2_, 1; CaCl_2_, 1.8; NaHCO_3_, 13.2; NaH_2_PO_4_, 1.8; and glucose, 11 (pH 7.4). This was previously described in [58]. Briefly, the freshly cut PMs horizontally mounted in a temperature-controlled chamber (30 ± 0.1 °C) were stretched to a length at which the tension of contraction was at its maximum. Stimuli were applied as rectangular pulses with a duration of 5 ms and an amplitude 25% higher than the excitation threshold, for which bipolar Ag-AgCl electrodes were used. Before each experiment, PMs were stimulated at a frequency of 0.3 Hz for 1 h until complete mechanical stabilization. Muscles that had autorhythmic activity, were mechanically unstable, or demonstrated a contractile force of less than 1 mN were excluded from the study. HDP-1 was dissolved in the Tyrode solution and added to the chamber to obtain the studied concentration. The following parameters were recorded: the time to 50 and 95% relaxation, the time to peak tension, and the force of contraction. In order to measure the force–frequency relationship (after complete mechanical stabilization), the stimulation rate was increased stepwise to 0.1, 0.2, 0.3, 0.4, 0.5, 0.6, 0.8, 1.0, 1.5, 2.0, and 3.0 Hz. The rest effect was studied at a stimulation frequency of 1 Hz. After each measurement, the PMs were paced until full mechanical recovery of the muscles.

### 5.4. Contractility of ARs

Aortas were cut from anesthetized rats, placed in a Tyrode solution (similar to that used for the PMs but with 2.5 mM CaCl_2_), and cleaned of loose connective tissue and fat. Rings of 2–3 mm were cut. A total of 2 to 4 ARs were obtained from one rat. The ARs were mounted horizontally on two tungsten wires. One wire was fixed to the wall of the organ chamber, and the other was connected to a force transducer to record the isometric tension. For the isometric tension recording, an instrument similar to that used for the PM studies was used. The only difference was the ADC/DAC board, with an E14-440 (L-Card) being installed for the AR investigation. The temperature of the Tyrode solution flowing through the tissue chamber at a flow rate of 1 mL/min was maintained at 30 ± 0.1 °C. After establishing the initial load of 2 g, the ARs were adapted for 60 min. After this period, the tension on the ARs was taken as the resting tension. To test the ability of the ARs to develop contractions, the rings were exposed to an isotonic depolarizing solution (with an equimolar replacement of NaCl to obtain 80 mM KCl in solution) for 20 min. After washing (40 min), 10 µM PE was added, and then after 30 min, 10 µM ACh was added to check the integrity of the endothelium. After 15 min, the ACh was washed out with Tyrode solution containing PE, and the tension returned to the PE level. At this point, HDP-1 in Tyrode solution with PE was applied. No HDP-1 was added to the control. Anywhere from 4 to 7 ARs were used for each measurement.

### 5.5. Data Analysis and Statistics

Student’s *t*-test was used to compare the continuous variables. One-way ANOVA with Dunnett’s post hoc test was used for multiple-group comparisons. A *p* value < 0.05 was predetermined as a statistically significant difference. All data are presented as the mean ± standard error (S.E.).

## Data Availability

All data obtained in this study are contained within the article.

## Supplementary Materials

The following supporting information can be downloaded at: https://www.mdpi.com/article/10.3390/toxins16020100/s1, Figure S1: Isolation of HDP-1 by cation-exchange chromatography under conditions described in [25]. Figure S2: High-resolution mass-spectra of HDP-1 registered in positive mode. Figure S3: Effect of different HDP-1 concentrations on the kinetic parameters of contraction. TPT, time to peak tension; TR 50%, the time to 50% relaxation; TR 95%, the time to 95% relaxation. No statistically significant differences with control were observed for all experiments. Table S1: Effect of different HDP-1 concentrations on the kinetic parameters of the papillary muscle contraction.
