# Supplementary material for: Effects of the Heterodimeric Neurotoxic Phospholipase A2 from the Venom of Vipera nikolskii on the Contractility of Rat Papillary Muscles and Thoracic Aortas
Source: Toxins (Basel). 2024 Feb 10;16(2):100. doi: 10.3390/toxins16020100 (PMC10891809; doi:10.3390/toxins16020100)
Supplement: Supplementary file 1 [file toxins-16-00100-s001.zip › toxins-2821263-supplementary.pdf]

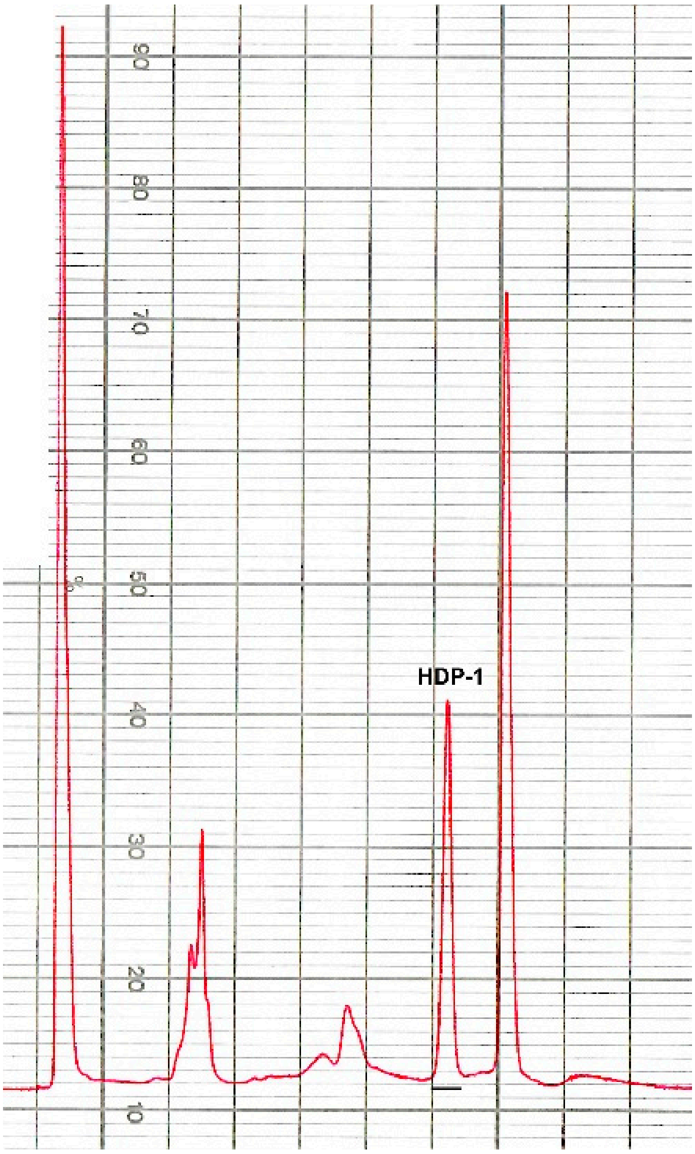

Figure S1. Isolation of HDP-1 by cation-exchange chromatography under conditions described in [25]. Horizontal bar indicates the fraction containing HDP-1.

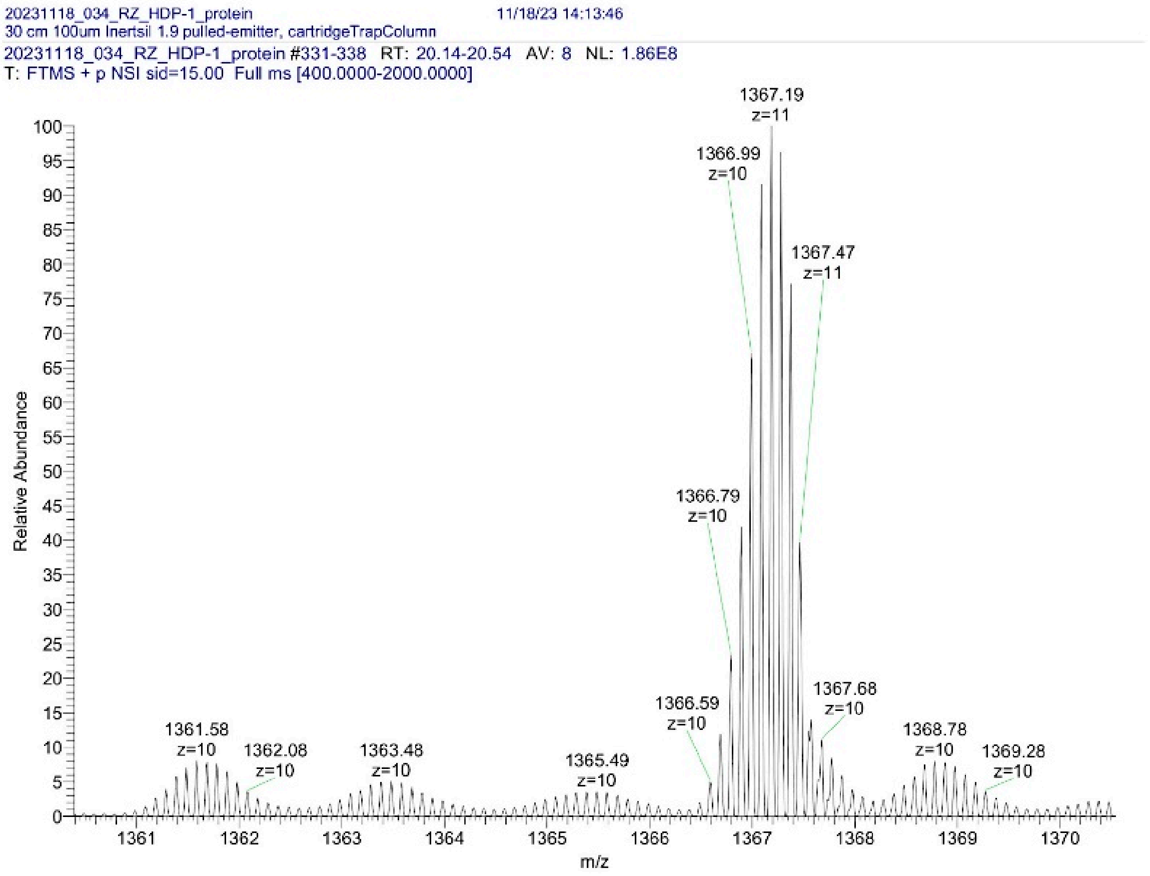

(a)

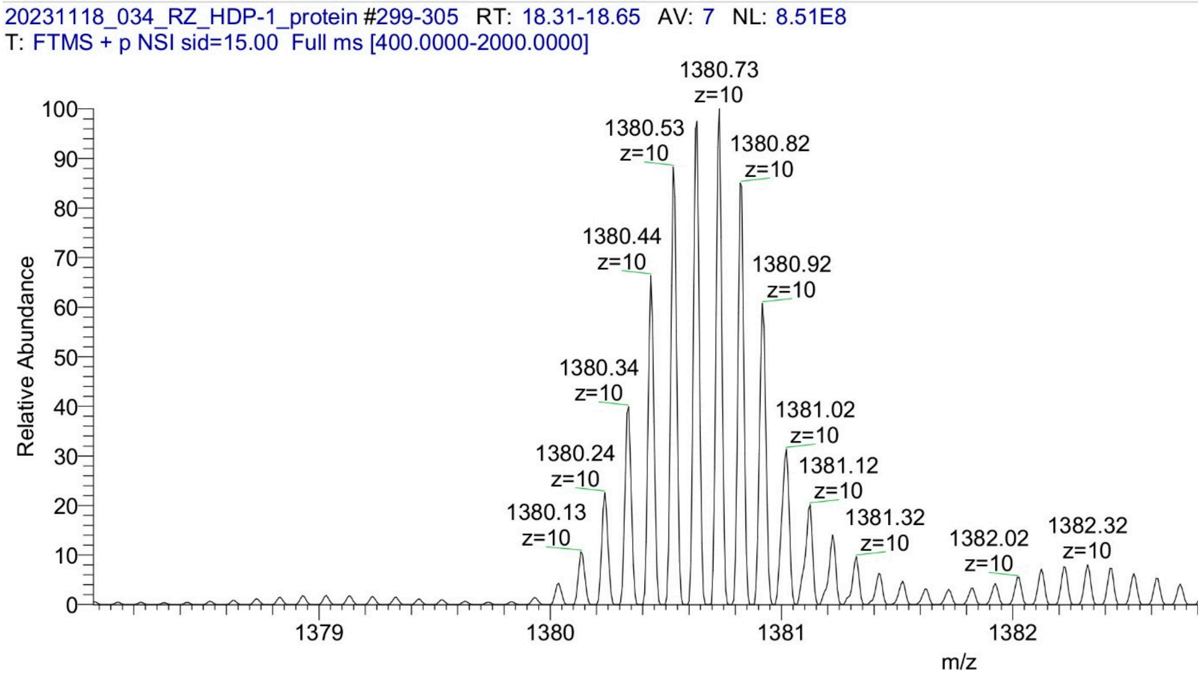

(b)

Figure S2. High-resolution mass-spectra of HDP-1 registered in positive mode. (a) Fragment of the spectrum of the acidic HDP-1 subunit (HDP-1I), z = +10. It should be noted that the experimental mass is 40 ae higher than the calculated one, which is apparently due to the bound calcium ion. A signal corresponding to a

protein without calcium could not be detected. (b) Fragment of the spectrum of the basic HDP-1 subunit (HDP-1P), z = +10. Within the experimental error, the determined mass corresponds to the calculated one.

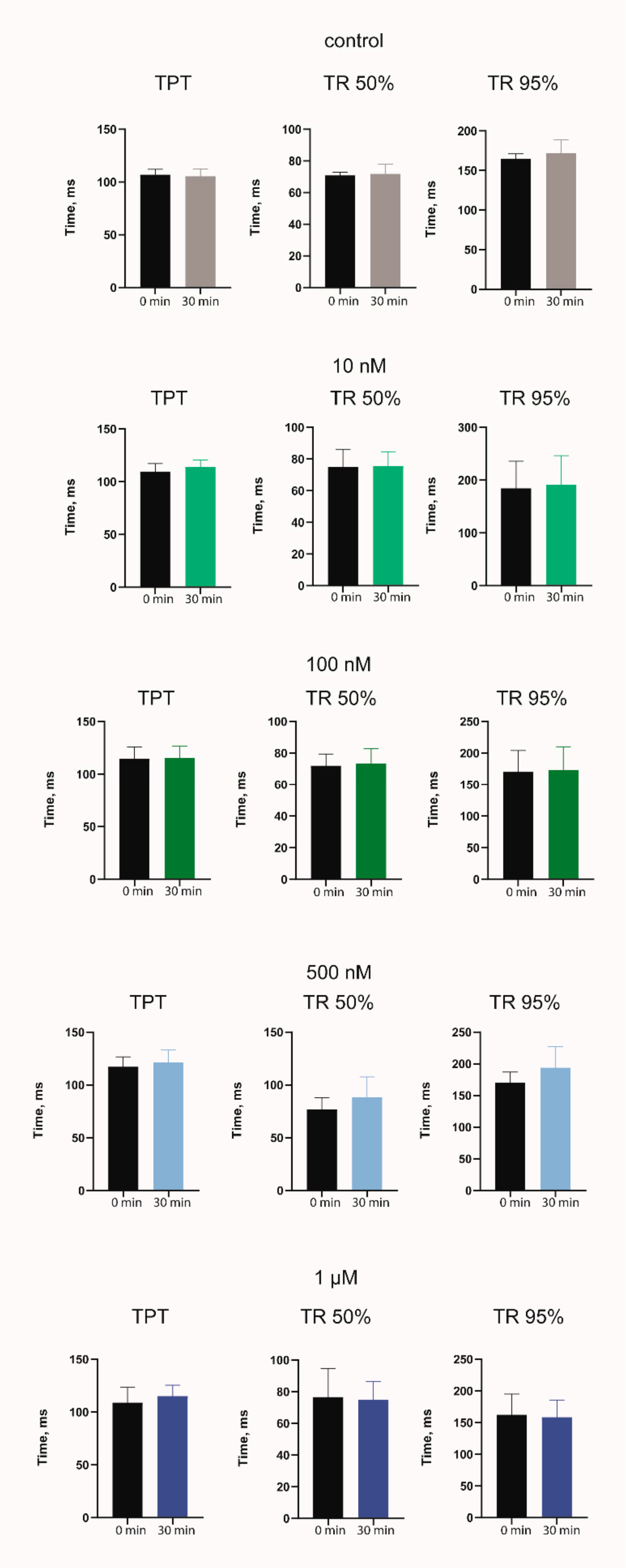

Figure S3. Effect of different HDP-1 concentrations on the kinetic parameters of contraction. TPT, time to peak tension, TR 50%, the time from peak tension to 50% relaxation, TR 95%, the time from peak tension to 95% relaxation. No statistically significant differences with control were observed for all experiments.

Table S1. Effect of different HDP-1 concentrations on the kinetic parameters of the papillary muscle contraction.

**Parameter**	**Control n=5**		**HDP-1 concentration**							
			**10nM n=5**		**100nM n=5**		**500nM n=6**		**1 μM n=6**	
	**Initial**	**30 min**	**Initial**	**30 min**	**Initial**	**30 min**	**Initial**	**30 min**	**Initial**	**30 min**
Time to peak tension, ms	107±2	105±3	110±3	114±3	115±5	116±5	119±6	123±5	109±6	115±4
Time of relaxation to 50%, ms	71±1	72±3	75±5	76±4	72±3	74±4	78±4	89±9	77±7	75±5
Time of relaxation to 95%, ms	165±3	172±8	185±23	191±25	170±15	173±16	167±7	186±11	162±13	158±11
